# Acute Disseminated Encephalomyelitis with Seizures and Myocarditis: A Fatal Triad

**DOI:** 10.3390/medicina56060277

**Published:** 2020-06-04

**Authors:** Hanne Lademann, Astrid Bertsche, Axel Petzold, Fred Zack, Andreas Büttner, Jan Däbritz, Christina Hauenstein, Erik Bahn, Christian Spang, Daniel Reuter, Philipp Warnke, Johannes Ehler

**Affiliations:** 1 Department of Pediatrics, Interdisciplinary Pediatric Intensive Care Medicine, University Medical Center Rostock, 18057 Rostock, Germany; hanne.lademann@med.uni-rostock.de; 2 Department of Pediatrics, Neuropediatrics, University Medical Center Rostock, 18057 Rostock, Germany; astrid.bertsche@med.uni-rostock.de; 3 Department of Neuroimmunology, The National Hospital for Neurology and Neurosurgery, Queen Square, UCL Institute of Neurology, London WC1N 3BG, UK; a.petzold@ucl.ac.uk; 4 Institute of Legal Medicine, University Medical Center Rostock, 18055 Rostock, Germany; fred.zack@med.uni-rostock.de (F.Z.); andreas.buettner@med.uni-rostock.de (A.B.); 5 Department of Pediatrics, University Medical Center Rostock, 18057 Rostock, Germany; jan.daebritz@med.uni-rostock.de; 6 Institute of Diagnostic and Interventional Radiology, Pediatric Radiology and Interventional Radiology, University Medical Center Rostock, 18057 Rostock, Germany; christina.hauenstein@med.uni-rostock.de; 7 Institute of Neuropathology, University Medical Center Göttingen, 37073 Göttingen, Germany; ebahn@gwdg.de; 8 Department of Anesthesiology and Intensive Care Medicine, Interdisciplinary Pediatric Intensive Care Medicine, University Medical Center Rostock, 18057 Rostock, Germany; christian.spang@med.uni-rostock.de (C.S.); daniel.reuter@med.uni-rostock.de (D.R.); 9 Institute of Medical Microbiology, Virology and Hygiene, University Medical Center Rostock, 18057 Rostock, Germany; philipp.warnke@med.uni-rostock.de

**Keywords:** delirium, critical ill patient, critical illness, ADEM

## Abstract

Autoimmune pathology of acute disseminated encephalomyelitis (ADEM) is generally restricted to the brain. Our objective is to expand the phenotype of ADEM. A four-year-old girl was admitted to the pediatric emergency room of a university medical center five days after a common upper respiratory tract infection. Acute symptoms were fever, leg pain, and headaches. She developed meningeal signs, and her level of consciousness dropped rapidly. Epileptic seizure activity started, and she became comatose, requiring intubation and mechanical ventilation. Serial brain magnetic resonance imaging (MRI) illustrated the fulminant development of ADEM. Treatment escalation with high-dose corticosteroids, immunoglobulins, and plasma exchange did not lead to clinical improvement. On day ten, the patient developed treatment-refractory cardiogenic shock and passed away. The postmortem assessment confirmed ADEM and revealed acute lymphocytic myocarditis, likely explaining the acute cardiac failure. Human metapneumovirus and picornavirus were detected in the tracheal secrete by PCR. Data sources–medical chart of the patient. This case is consistent with evidence from experimental findings of an association of ADEM with myocarditis as a postinfectious systemic autoimmune response, with life-threatening involvement of the brain and heart.

## 1. Introductions

Acute disseminated encephalomyelitis (ADEM) is a frequent postinfectious disease in children, with a good prognosis [1]. The systemic symptoms include headaches (~60%), fever (~40%), and meningism (~30%). Seizures are rarer (~17%) and typically associated with febrile illness [1]. Neurological signs are polysymptomatic, with bilateral loss of vision, cranial neuropathies, altered mental state and level of consciousness, and pyramidal signs. Less frequent are extrapyramidal signs and sensory disturbances. Cardiac complications have only been rarely reported [2,3]. Myocarditis has not yet been described in humans but is known to coexist with ADEM and seizures in the Theiler mouse model of a demyelinating disease [4]. Here, we report the first human case of this triad. The study has been approved by the Ethics Committee of the Medical Faculty of the University of Rostock, Germany (Approval No.: A 2020-0045 at the 26 February 2020).

## 2. Case Report

**Figure 1:**
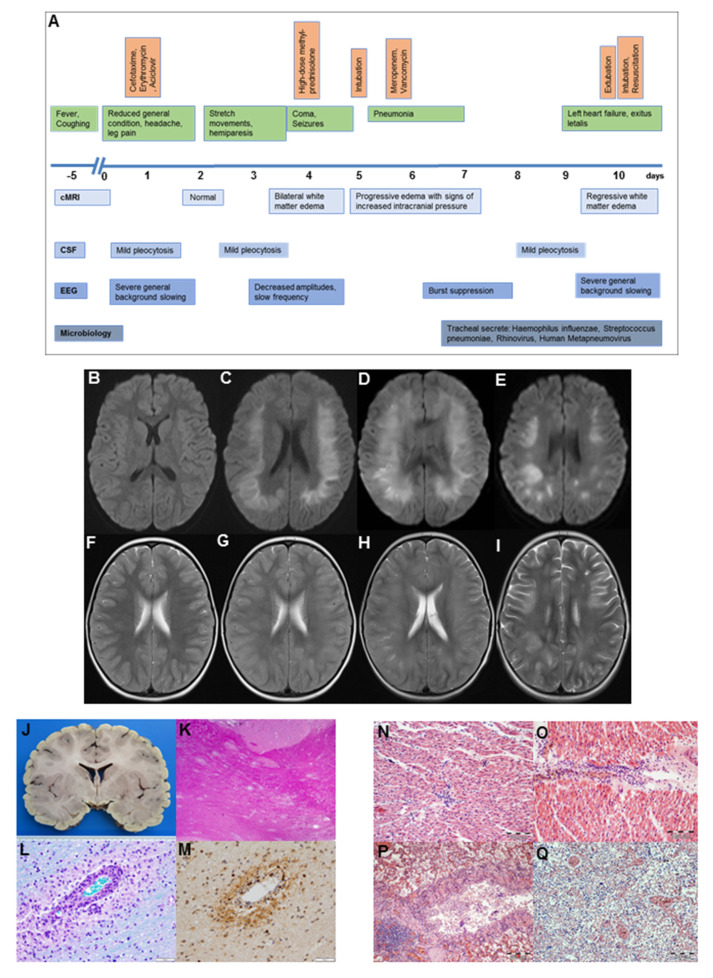
(**A**): Overview of the clinical course (green), as well as medical treatment (orange) and diagnostic findings (blue). (**B**–**I**): Cerebral magnetic resonance imaging of a 4-year old patient. Axial diffusion-weighted (**B**–**E**) and T2-weighted (**F**–**I**) imaging representing normal results on day 2 (**B**,**F**). On day 4, severe bilateral white matter cytotoxic and vascular edema without contrast medium enhancement are visible (**C**,**G**). Repeated cMRIs at day 5 represent a further progression of white matter edema with signs of elevated intracranial pressure (**D**,**H**), whereas a cMRI at day 10 gives evidence for a regression of the white matter edema (**E**,**I**). (**J**–**M**): Neuropathology examination. The macroscopic horizontal brain section demonstrates no visible abnormalities (**J**). Disseminated areas of perivenous demyelination in the white matter of the temporal lobe, demonstrated by haematoxylin and eosin (**K**, 20×) and LFB-PAS staining (**L**, 200×). Foamy perivenous macrophages (**L**) and scattered CD3-positive T cells (**M**) dominate the inflammatory infiltrate (200×). (**N**–**P**): Histology of myocardium and lung. Myocardium with interstitial lymphocytic infiltration, haematoxylin and eosin staining (**N**, 40×; **O**, 40×). Lung tissue with lymphocytic bronchitis and peribronchitis, haematoxylin and eosin staining (**P**, 20×), and haemorrhagic pulmonary oedema of the lungs, haematoxylin and eosin staining (**Q**, 20×). cMRI, cerebral magnetic resonance imaging; CSF, cerebrospinal fluid; EEG, electroencephalography; TPE, therapeutic plasma exchange.

A 4-year-old girl was admitted to the emergency room with headaches and bilateral leg pain. The history was taken from her mother, who reported a five-day history of a febrile (40 °C, 104 °F) cough. The girl was born healthy after an uncomplicated pregnancy. Her past medical history included asthma and allergy to house dust mites. She had had routine vaccinations except for rotavirus. On examination, there were no additional symptoms or signs. The girl was admitted to the general pediatric ward for further observation and symptomatic treatment. The next day, her clinical condition deteriorated with meningeal signs and a reduced level of consciousness (Figure 1A). The patient was transferred to the pediatric intensive care unit with suspected community-acquired meningoencephalitis, and a cerebrospinal fluid (CSF) examination was performed, demonstrating a mild pleocytosis (Table 1).

Antibiotic (200 mg/kg/day of cefotaxime and 50 mg/kg/day of erythromycin) and antiviral therapy (45 mg/kg/day of acyclovir) were immediately started, and cerebral magnetic resonance imaging (cMRI) was performed. This MRI did not reveal any pathological findings (Figure 1B,F). Laboratory examinations from blood and CSF did not show any pathogens. Despite antimicrobial therapy, the patient’s condition rapidly deteriorated, with a reduced level of consciousness up to coma (Glascow’s coma scale of 4), accompanied by right-sided hemiparesis, generalized seizures, and abnormal flexion and extension movements of her extremities to pain stimuli. On day 4, cMRI revealed a severe bilateral white matter cytotoxic and vascular edema without contrast medium enhancement (Figure 1C,G). Electroencephalography (EEG) demonstrated severe general background slowing without specific seizure patterns. Acute disseminated encephalomyelitis (ADEM) was suspected, and treatment with high-dose methylprednisolone (20 mg/kg/day) was started. Repeated CSF analysis confirmed a mild pleocytosis (Table 1), but no pathogens were detected despite extensive microbiologic diagnostics (Table S1). Furthermore, blood examinations did not reveal any pathogens. A panel of autoimmune and paraneoplastic antibodies (Ab), among others, neuronal and Ganglioside-Ab, alpha feto protein, and myelin oligodendrocyte glycoprotein (MOG)-Ab, was negative. Due to the rapid deterioration of the patient’s condition, intravenous immunoglobulin treatment (2 g/kg/day) was started. Neurological status progressively declined, and gas exchange further deteriorated on the basis of pneumonia. Thus, the patient had to be intubated and mechanically ventilated at day 5. Repeated brain MRIs on day 5 emphasized a further progression of white matter edema and suspected elevated intracranial pressure (Figure 1D,H). External ventricular drainage was implemented and a brain biopsy was taken. Therapy was escalated with four single-sessions of therapeutic plasma exchange (70 mL/kg/day) between days 5 and 9.

Despite deep sedation with midazolam (0.15 mg/kg/h) and analgesia with remifentanil (0.2 µg/kg/min), the patient presented generalized seizures. Treatment with levetiracetam (40 mg/kg/day) was initiated. Due to insufficient anticonvulsive effects, this was supplemented with phenobarbital (20 mg/kg/day). The only pathological results were for rhinovirus, human metapneumovirus, haemophilus influence, staphylococcus aureus, and streptococcus pneumoniae in the tracheal secretion on day 7 by polymerase chain reaction (PCR). Otherwise, repeated extensive screening for viral, fungal, and bacterial pathogens, including rare agents, was performed in CSF, blood, stool, urine, and brain biopsy material, with no causative findings (Table S1). The patient was treated with a combination of meropenem (120 mg/kg/day) and vancomycin (60 mg/kg/day). She remained hemodynamically stable with minimal inotropic support (norepinephrine </= 0.03 µg/kg/min; dobutamine </= 3 µg/kg/min.

At day ten, brain MRI examination presented the first evidence of regressive white matter edema in the central nervous system (CNS; Figure 1E,I). EEG still showed signs of a general background slowing. As she became responsive and was seemingly able to protect her airways upon the reduction of sedation, she started to be weaned from ventilation. Rapidly after extubation, the patient developed massive pulmonary edema with foamy secretions and severe hypoxemia. The patient was immediately reintubated, and a bronchoscopy was performed. In parallel, the patient became hemodynamically and increasingly unstable, with the need for high doses of catecholamines. The first episode of cardiac arrest was resolved by short resuscitation. Emergency transthoracic echocardiography revealed acute signs of left heart failure. Left ventricular ejection fraction was quantified with 38%, and a grade 2 mitral and tricuspid insufficiency was recognized. The patient’s condition further and rapidly deteriorated, with persistent severe hypoxemia, progressive left heart failure, and insufficient response to high-dose catecholamine therapy with up to 2 µg/kg/min of epinephrine. There was no evidence for endocarditis on TTE. Treatment with veno-arterial extracorporal membrane oxygenation (ECMO) was discussed within an interdisciplinary team and the parents. However, because of the extent of the brain injury and an unlikely favorable outcome, ECMO was not started. Briefly afterwards, the girl passed away.

The independent histopathological examination of the brain in the Institute of Legal Medicine Rostock, Germany, and in one of the German reference centers for Neuropathology (University Hospital Göttingen, Germany) confirmed the diagnosis of ADEM (Figure 1J–M). Antibodies against MOG in blood serum and CSF were negative. In addition, the autopsy provided evidence for a disseminated viral infection causing massive pulmonary and cardiac infiltration of lymphocytic (Figure 1N–Q). Postmortem diagnosis of viral myocarditis was made. This was the most likely reason for the acute cardiac failure. There was no evidence for endocarditis.

In order to identify an alternative causative pathogen, a nested-PCR diagnostic was performed from postmortem lung, brain, and heart tissues, but no pathogen was detected (Table S1). As the case preceded the COVID19 pandemic, and all samples had been used up, we were unable to test for coronaviruses.

## 3. Discussion

We present the first human case with a triad of ADEM, myocarditis, and seizures in a pediatric patient. The onset was a febrile upper respiratory tract infection five days before admission. ADEM is a multifocal inflammatory demyelinating disorder of the CNS that usually occurs one to two weeks after viral or bacterial infection [1]. An autoimmune-mediated reaction against myelin-containing proteins is discussed [5].

A recent metaanalysis described an overall good long-term outcome after ADEM [6]. Nevertheless, fever on admission, ventilator-associated pneumonia, and meningism, as seen in our patient, seem to be associated with a poor outcome [7]. So far, only the acute hemorrhagic variant of leukoencephalitis (AHLE) has been described as a fulminant variant of ADEM, which occurs in only 2% of cases and is also associated with a poor outcome [8,9,10]. 

Moreover, histopathological examination specified a myocarditis, which is also an inflammatory disease caused by microbial infections or autoimmunity [11]. As in ADEM, the persistence of a virus or a postviral (auto)immune process can merge into chronic (myocardial) tissue damage [9]. Hence, in our patient, an overwhelming immune response linked to common feverish colds may have been causative for tissue damage in the heart and in the brain.

In addition, neurocardiogenic injury has been described in the literature [12]. It is usually observed in patients with subarachnoid hemorrhage, stroke, and after meningitis [13]. Severe stress, as experienced by our patient in the context of a severe CNS infection, affects the myocardium through the sympathetic nervous system [14]. We initially suspected that neurocardiogenic mechanisms caused left heart failure. In contrast to our assumptions, the postmortem histopathological examination of the heart did not give evidence for typical necrosis of the myocardium but demonstrated massive lymphocytic infiltrates, indicating likely viral myocarditis. Microbiological analysis of the patient’s tracheal secretion showed different bacteria of the commensal flora, which are not regarded as causative and streptococcus pneumoniae, which had been covered by antibiotic therapy. Furthermore, two potentially causative agents were detected: human metapneumovirus, which can be associated with encephalitis, focal seizures, and status epilepticus [15], and rhinovirus, a respiratory pathogen and member of the Picornaviridae family. In mice, but not humans, a virus of the same family (Theiler’s murine encephalomyelitis virus, TMEV) not just induces encephalomyelitis, but also cardiac lesions [16]. This virus triggers innate immunity as well as antiviral immune responses and autoimmune-induced myocardial damage [16]. We would, however, be hesitant to claim a definite link between rhinovirus and myocarditis because of the extraordinary rarity of this association [17,18]. Nevertheless, this raises the question if certain viruses can induce similar injury to the brain and heart in humans, too. Different viruses were found to be associated with either multiple sclerosis, myocarditis, or epilepsy [4]. Extended diagnostics were performed on blood, serum, postmortem lung, brain, and heart tissues, but neither pathogens nor antibodies or evidence for paraneoplastic cause could be found (Table S1). An unknown pathogen could have triggered the disease.

## 4. Conclusions

In conclusion, the patient died from postinfectious acute heart failure caused by a viral myocarditis in the context of fulminant ADEM. This is to our knowledge the first report of a fatal triad of ADEM, seizures, and myocarditis in humans. The detection of the picornavirus suggests a simultaneous autoimmune response of the heart and brain, as demonstrated experimentally [16].

## Figures and Tables

**Table 1:** Results from routine blood and cerebrospinal fluid examinations between days 1 and 10.

		1	2	3	5	7	8	9	10 *
Blood									
White cell count	10^9^/L	22	19	9	20	15	22	19	39
CRP	mg/L	9	12		7	4.55	<1	<1	<1
PCT	ng/mL	0.06	0.06	0.06	0.4	0.06	0.06	0.07	0.43
CSF									
White cell count	10^9^/L	97		95					94
Protein level	mg/L	565		289					290
Lactate level	mmol/L	2.7		2.0					2.5
Glucose level	mmol/L	2.7		4.5					4.5

CRP–c-reactive protein, CSF-cerebrospinal fluid, PCT-procalcitonin * before resuscitation.

## Supplementary Materials

The following are available online at https://www.mdpi.com/1010-660X/56/6/277/s1, Table S1: Diagnostics.
