# Supplementary material for: Acute Disseminated Encephalomyelitis with Seizures and Myocarditis: A Fatal Triad
Source: Medicina (Kaunas). 2020 Jun 4;56(6):277. doi: 10.3390/medicina56060277 (PMC7353866; doi:10.3390/medicina56060277)
Supplement: Supplementary file 1 [file medicina-56-00277-s001.pdf]

Table S1. Diagnostics.

Bone-marrow puncture: inconspicious

Autoimmunological disorders
ANA
c-ANCA
AK against PR3
p-ANCA
Myeloperoxidase
Complement C3
Complement C4
Total complement activity CH50
Neuronal ab (CSF, serum)
Ganglioside ab (CSF, serum)
TSH receptor ab

Paraneoplastic disorders
Alpha-fetoprotein
Beta-HCG

negative

Microbiology
Human herpesvirus PCR test
(including HHV-1, -2, -3, -4, -5, -6)

Serology:
Borrelia burgdorferi: IgM and IgG
C. pneumoniae: IgA and IgG
C. psittaci: IgA and IgG
C. burnetii: IgM and IgG
M. pneumoniae: IgG borderline test result, IgM and IgA
L. pneumophila: IgM and IgG
TPHA
Toxoplasmose
C. jejuni: IgA and IgG
Yersinia: IgM, IgA and IgG
Leptospira IgM and IgG
Hantavirus: IgM and IgG
Sandfly fever virus: IgM and IgG
Rubulavirus: IgG positive, IgM
Morbilli virus: IgG positive, IgM
FSME virus: IgM and IgG, IgG ab Liquor

without

Rubella virus: IgG positive, IgM
HIV 1/2 ab + p24Ag
QuantiFERON TB gold plus test: not determinable

Serum antibodies (IIFT)

Borna disease: IgM/ IgG
Sandfly fever virus
Usutu virus: IgM/ IgG
Rickettsia typhi
Naegleria fowleri
Rabies virus
16S rDNA PCR
18S rDNA PCR

negative

CSF

Meningitis-multiplex-PCR (BIOFIRE FILMARRAY ME Panel
Human herpesvirus PCR test
(including HHV-1, -2, -3, -4, -5, -6)
16S rDNA PCR
Borrelia
JC virus DNA
Borrelien-Serology
18S PCR
Flavivirus
VSBV-1 (variegated Squirrel Bornavirus)
BoDV-1 (Borna Disease)

negative

Anti-Neuronal Antibodies IgG (IFT)

Anti

- -Aquaporin-4
- -Glutamat-Rezeptors (Typ NMDA)
- -CASPR2
- -Hu
- -Ri
- -ANNA-3
- -Yo
- -Tr/DNER
- -Myelin
- -Ma/ta
- -GAD65
- -Amphysin
- -GABA-b-Rezeptors
- -LGI1
- -CARPVIII
- -Glycin-Rezeptors
- -mGluR1
- -mGluR5
- -GABA-a-Rezeptors
- -Rho GTPase activating protein 26
- -Recovirus
- -GluRD2
- -Flotilin
- -ITPR1
- -Homer3
- -Neurochondrin

negative

Nested-PCR from lung, heart and brain tissue

Echovirus
Parvovirus B19
Humane Herpes virus 6, 7, 8
Epstein Barr Virus
Adenovirus
Human Cytomegalovirus
Herpes simplex 1 and 2 virus
Varizella zoster virus
Toxoplasma gondii
Borrelia burgdorferi

negative

Abbreviations: CSF = cerebrospinal fluid, ab = Antibodies, Ag = antigene
